# Preparing for future pandemics: frailty associates with mortality in hospitalised older people during the entire COVID-19 pandemic, a Dutch multicentre cohort study

**DOI:** 10.1007/s41999-024-01001-1

**Published:** 2024-06-07

**Authors:** Bas F. M. van Raaij, Raymond Noordam, Rosalinde A. L. Smits, Veerle M. G. T. H. van der Klei, Steffy W. M. Jansen, Carolien M. J. van der Linden, Harmke A. Polinder-Bos, Julia Minnema, Lisanne Tap, Jessica M. van der Bol, Esther M. M. van de Glind, Hanna C. Willems, Floor J. A. van Deudekom, Rikje Ruiter, Barbara C. van Munster, Sarah H. M. Robben, Henrike J. Schouten, Dennis G. Barten, Jacinta A. Lucke, Geeske Peeters, Stella Trompet, Yvonne M. Drewes, Frederiek van den Bos, Jacobijn Gussekloo, Simon P. Mooijaart, the COOP study group, Simon P Mooijaart, Harmke A Polinder-Bos, Karel G. M Moons, Maarten Smeden, Geeske Peeters, René J. F Melis, Petra J. M Elders, Jan Festen

**Affiliations:** 1 Department of Internal Medicine, Section of Geriatrics and Gerontology, Leiden University Medical Center, Albinusdreef 2, 2333ZA Leiden, The Netherlands; 2 LUMC Center of Medicine for Older People, Leiden University Medical Center, Leiden, The Netherlands; 3 Department of Geriatric Medicine, Catharina Hospital, Eindhoven, The Netherlands; 4 Division of Geriatric Medicine, Department of Internal Medicine, Erasmus MC, University Medical Center Rotterdam, Rotterdam, The Netherlands; 5 Department of Geriatric Medicine, Reinier de Graaf Hospital, Delft, The Netherlands; 6 Department of Geriatric Medicine, Alrijne Hospital, Leiderdorp, The Netherlands; 7 Department of Internal Medicine and Geriatrics, Amsterdam University Medical Center, Amsterdam, The Netherlands; 8 Department of Geriatric Medicine, OLVG Hospital, Amsterdam, The Netherlands; 9 Department of Internal Medicine, Maasstad Hospital, Rotterdam, The Netherlands; 10 Department of Geriatric Medicine, University Medical Center Groningen, Groningen, The Netherlands; 11 Department of Geriatric Medicine, Elisabeth-TweeSteden Hospital, Tilburg, The Netherlands; 12 Department of Geriatric Medicine, Gelre Hospital, Apeldoorn, Zutphen, The Netherlands; 13 Department of Emergency Medicine, VieCuri Medical Center, Venlo, The Netherlands; 14 Department of Emergency Medicine, Spaarne Gasthuis, Haarlem, the Netherlands; 15 Department of Geriatric Medicine, Radboud University Medical Center, Nijmegen, The Netherlands; 16 Department of Public Health and Primary Care, Leiden University Medical Center, Leiden, The Netherlands

**Keywords:** Frailty, COVID-19, In-hospital mortality

## Abstract

**Purpose:**

Viral mutations and improved prevention or treatment options may have changed the association of frailty with mortality throughout the COVID-19 pandemic. We investigated how associations of frailty with in-hospital mortality changed throughout the pandemic in older people hospitalised for COVID-19.

**Methods:**

The COVID-OLD study included COVID-19 patients aged ≥ 70 years hospitalised during the first (early 2020), second (late 2020), third (late 2021) or fourth wave (early 2022). Based on the clinical frailty scale, patients were categorised as fit (1–3), pre-frail (4–5) or frail (6–9). Associations of frailty with in-hospital mortality were assessed with pairwise comparisons with fit as reference category and modelled using binary logistic regression adjusted for age and sex.

**Results:**

This study included 2362 patients (mean age 79.7 years, 60% men). In the first wave, in-hospital mortality was 46% in patients with frailty and 27% in fit patients. In-hospital mortality decreased in each subsequent wave to 25% in patients with frailty and 11% in fit patients in the fourth wave. After adjustments, an overall higher risk of in-hospital mortality was found in frail (OR 2.26, 95% CI: 1.66–3.07) and pre-frail (OR 1.73, 95% CI: 1.27–2.35) patients compared to fit patients, which did not change over time (*p* for interaction = 0.74).

**Conclusions:**

Frailty remained associated with a higher risk of in-hospital mortality throughout the entire COVID-19 pandemic, although overall in-hospital mortality rates decreased. Frailty therefore remains a relevant risk factor in all stages of a pandemic and is important to consider in prevention and treatment guidelines for future pandemics.

**Supplementary Information:**

The online version contains supplementary material available at 10.1007/s41999-024-01001-1.

## Key summary points

**Aim:**

We aim to investigate how associations of frailty with in-hospital mortality changed throughout the pandemic in older people hospitalised for COVID-19.

**Findings:**

Older hospitalised COVID-19 patients with frailty had a higher in-hospital mortality risk over the entire course of the pandemic. Older hospitalised COVID-19 patients had a lower in-hospital mortality risk in each subsequent wave, which reflects the effects of improved prevention and treatment options.

**Message:**

Frailty is a relevant risk factor in all stages of a pandemic, which indicates that frailty is important to consider in prevention and treatment guidelines for future pandemics.

**Supplementary Information:**

The online version contains supplementary material available at 10.1007/s41999-024-01001-1.

## Introduction

During the first two years of the pandemic, approximately one-third of patients hospitalised for COVID-19 in the Netherlands was aged 70 years or older, while they only make up one-seventh of the population [1]. The high rate of hospitalisations placed a high burden on the healthcare systems with a significant pressure on hospital bed occupancy. Older age and frailty were risk factors for severe infection and mortality, both contributing to the large proportion of older people in the number of hospitalisations [2, 3]. Frailty is characterised by decreased physiological reserve and increased biological vulnerability to internal and external stressors [4]. It is currently unknown if the occurrence of viral mutations, improved diagnosis and treatment options and the introduction of vaccinations altered the association of frailty with in-hospital mortality over the course of the pandemic.

Since the start of the COVID-19 pandemic, different government policies were introduced in the Netherlands to prevent and reduce the spread of infection, improved diagnostics, treatment options and vaccinations became available and different viral variants arose. Policies regarding protection of public health introduced by the Dutch government included the vaccination program, a lockdown with limitation of contact moments, obligation to wear a facemask in public places and a variety of hygienic rules. The Dutch vaccination program for COVID-19 started in January 2021 with initial priority for older people due to limited initial supply. In addition to vaccinations, important new treatment options incorporated in treatment guidelines were dexamethasone and interleukin 6 (IL-6) inhibitors. The severe acute respiratory syndrome coronavirus 2 (SARS-CoV-2) mutated rapidly since the start of the pandemic which led to different viral variants [5, 6]. Viral mutagenesis increases efficiency of viral transmission, cell tropism and evasion of recognition by the immune system, all which enhance pathogenicity [7]. It is currently unknown to what extent all these factors affected the association of frailty with in-hospital mortality and disease severity of older people hospitalised for COVID-19 over the course of the pandemic. Understanding these associations may provide insights in the benefits of prevention, timely hospitalisation and treatment in frail older people, which is relevant for guidelines in future pandemics.

Therefore, we aim to investigate how associations of frailty with in-hospital mortality in hospitalised older people changed over the course of the COVID-19 pandemic.

## Methods

### Study design

The COVID-OLD study [8, 9], a retrospective multicentre cohort study, included older people hospitalised for COVID-19 during one of the four pandemic waves between February 2020 and April 2022 in the Netherlands. The 19 participating hospitals are listed in Appendix 1 and the number of inclusions per hospital for each pandemic wave are shown in Appendix 2. The necessity for formal approval of the study was waived by each local medical ethics committee, as data collection followed routine clinical practice. This study was conducted in accordance with the Declaration of Helsinki and Good Clinical Practice guidelines.

### The COVID-19 pandemic in the Netherlands

**Fig. 1:**
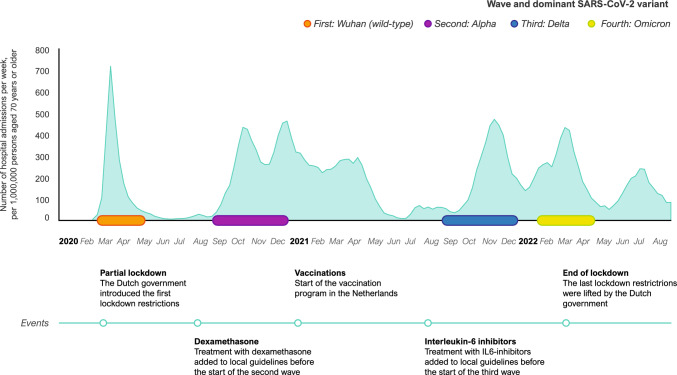
Timeline of hospital admissions for COVID-19 of older people (70 years and older) in the Netherlands in relation to inclusions of the COVID-OLD study and important events

We identified four pandemic waves based on peaks in hospital admission rates of patients aged 70 years and older, viral variants and important treatment implementations (Fig. 1). Occurrence of SARS-CoV-2 variants was evaluated by the National Institute for Public Health and the Environment Ministry of Health (Rijksinstituut over Volksgezondheid en Milieu, RIVM), in the Netherlands [1]. The waves were: first (February 27–May 15, 2020 [wild-type/Wuhan variant-dominant]), second (September 1–December 31, 2020 [Alpha variant-dominant]), third (September 1–December 31, 2021 [Delta variant-dominant]) and fourth (February 1–April 30, 2022 [Omicron variant-dominant]).

Each of the participating hospitals had a specific treatment protocol for COVID-19 of which most followed the treatment recommendations from the national guideline of the Dutch Working Party on Antibiotic Policy (Stichting Workgroups AntibioticaBeleid, SWAB). These recommendations changed over time as evidence changed and new treatment options emerged. The Dutch vaccination program for COVID-19 started on 6 January 2021, shortly after the end of the second wave and well before the start of the third wave. In other words, all patients included in this study had the opportunity of being vaccinated in the third and fourth wave. The vaccination coverage of the first series of COVID-19 vaccinations in people aged 70 years and older ranged from 92 to 93% in the Netherlands [10].

### Patient population

Patients aged 70 years and older were eligible for inclusion if they were admitted during one of the four defined waves and were not transferred from another hospital, because transferred patients had incomplete data. Patients with another primary diagnosis than COVID-19 at the time of admission,

defined as a polymerase chain reaction (PCR) test conducted > 1 day after admission, were excluded, because they possibly lacked a COVID-19-related indication for hospitalisation. In addition, patients discharged to another hospital and patients in whom both a trained researcher and geriatrician could not retrospectively determine the frailty status were excluded because their data were incomplete. Diagnosis of COVID-19 was confirmed with a positive PCR test result from a nasal or oropharyngeal swab in all patients except for patients in the first wave when PCR was not always rapidly available. The included patients in the first wave without a positive PCR test result were diagnosed based on symptoms, radiological abnormalities and laboratory findings.

### Data collection

Demographics, patient and clinical characteristics and outcomes of hospital treatment were collected from electronic medical records. Clinical characteristics included vital signs and laboratory findings on the day of admission as indicators of disease severity. In-hospital mortality was defined as patients who were deceased during admission or discharged to a hospice. Comorbidity was assessed with the Charlson Comorbidity Index (CCI) [11]. Frailty was assessed with the use of the clinical frailty scale (CFS) and patients were categorised in three groups: fit (CFS 1–3), pre-frail (CFS 4–5) or frail (CFS 6–9) [12].

The CFS was recorded during the first day of admission in hospitals that routinely collect frailty according to local guidelines. Otherwise, the CFS was recorded retrospectively by trained researchers based on available data in medical records. Retrospective assessment of CFS has been clinically validated in multiple studies [13, 14]. The researchers were trained by geriatricians and consultations were held in case of ambiguities. The classification of CFS was considered missing if both the researcher and geriatrician were unable to assess frailty.

### Statistical analysis

Continuous data are presented with mean and standard deviation (SD) or with median and interquartile range (IQR) depending on the normality of distribution. Categorical data are presented with absolute values and percentages. The associations of frailty with in-hospital mortality and disease severity were analysed for each different wave with pairwise comparisons with a fit classification as reference category to a pre-frail or frail classification. Differences of patient characteristics based on frailty were analysed with an identical method. These pairwise comparisons were performed with an independent t test or Mann–Whitney *U* test for continuous data depending on the normality of distribution. Categorical data were analysed with a chi-square test or Fisher’s exact test depending on the number of expected counts. To explore whether missingness of CFS was random or specific to a given subgroup of older people, we compared baseline characteristics of patients with a recorded CFS to patients with CFS missing. To evaluate whether associations with frailty and indicators of disease severity and outcomes changed over time, linear and binary logistic regressions were performed with an interaction term between CFS and wave (acting as a time variable). All regression models were corrected for age and sex.

The reported p values are two-sided and values less than 0.05 were considered statistically significant. Statistical analyses were performed with IBM SPSS Statistics, version 25.

## Results

**Fig. 2:**
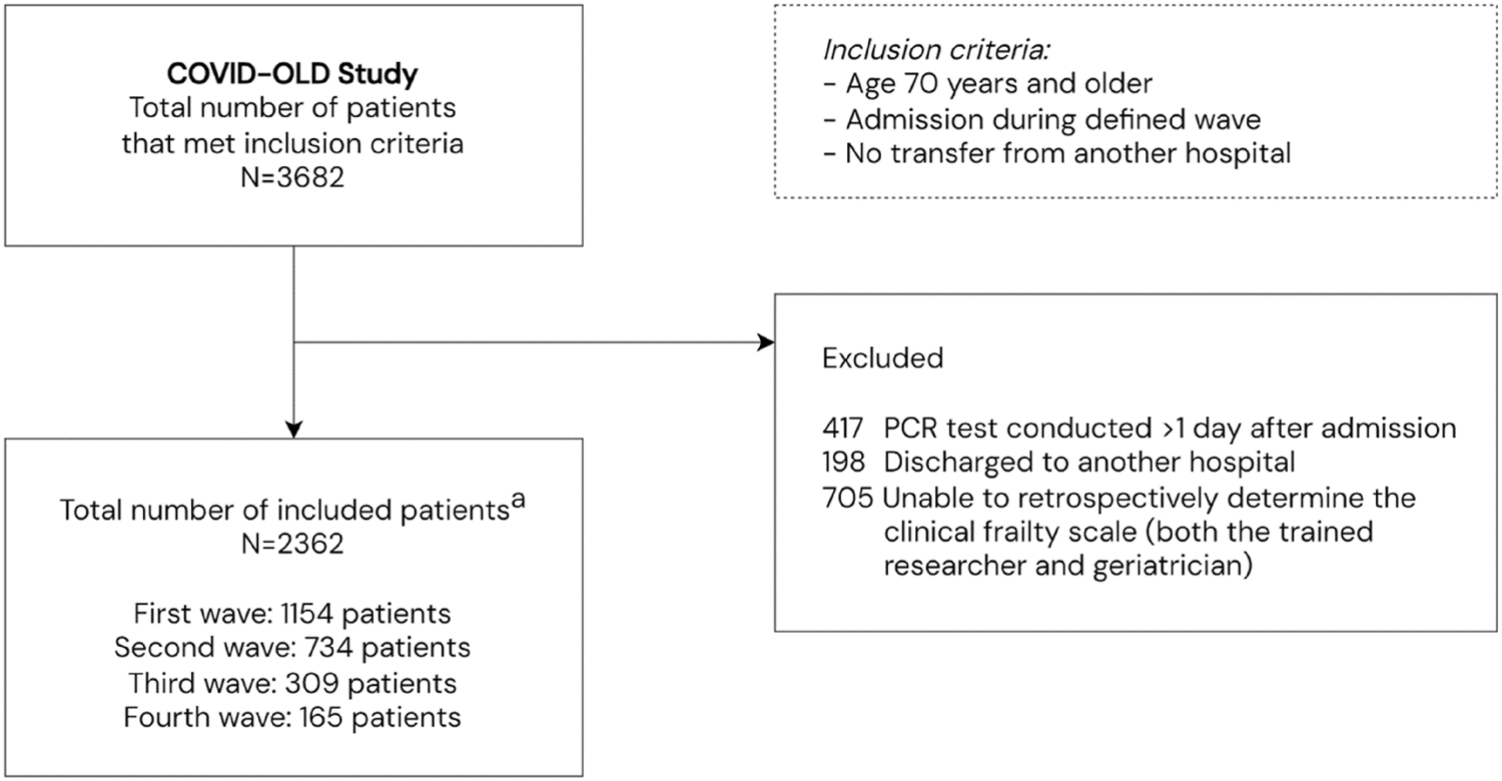
Flowchart of the COVID-OLD study. Number of participating hospitals in the different waves: 19 first wave, 10 second wave, 5 third wave and 4 fourth wave

In total, 3682 patients hospitalised for COVID-19 met the inclusion criteria (Fig. 2). We excluded 417 (11%) patients who acquired COVID-19 after admission, 198 (5%) patients discharged to another hospital and 705 (19%) patients with a missing frailty status. The remaining 2362 (64%) patients were included in the statistical analyses. A comparison of baseline characteristics between patients with a recorded frailty status that were included in this study and patients with a missing frailty status that were excluded is provided in Appendix 3. Overall, there were no relevant differences between patients with a recorded frailty status and a missing frailty status.

Baseline characteristics stratified by pandemic wave are listed in Table 1. The median age of patients was 79 (IQR 75–84) years and 60% were men. The mean BMI was 26.9 (SD 5.0) kg/m^2^ and 90% of patients lived independently before admission. The median CCI was 2 (IQR 1–3) points and 28% of patients had a history of lung disease. In the first wave, 519 (45%) patients were classified as fit (CFS 1–3), 304 (26%) patients as pre-frail and 331 (29%) patients as frail (CFS 6–9). The percentage of frail patients was higher in the fourth wave (41%) and the percentage of pre-frail patients was higher in the third (34%) and fourth wave (33%) (*p* < 0.001).

**Table 1:** Baseline characteristics of all patients and patients stratified by COVID-19 wave

Characteristic	All patients (*N* = 2362)	Wave			
		First (*N* = 1154)	Second (*N* = 734)	Third (*N* = 309)	Fourth (*N* = 165)
Age, years	79 [75–84]	79 [74–84]	80 [75–85]	79 [74–85]	79 [75–84]
Sex, men	1421 (60)	689 (60)	446 (61)	194 (63)	92 (56)
BMI, kg/m^2^	26.9 ± 5.0	27.2 ± 4.7	26.8 ± 5.1	26.7 ± 5.4	26.1 ± 5.3
Living situation					
Home	2075 (90)	1008 (90)	641 (88)	278 (92)	148 (93)
Nursing home	220 (10)	103 (9)	84 (12)	23 (8)	10 (6)
Other	24 (1)	15 (1)	7 (1)	0 (0)	2 (1)
Vaccinated*	290 (77)	NA	NA	212 (79)	78 (74)
Clinical frailty scale					
Fit, 1–3	986 (42)	519 (45)	313 (43)	110 (36)	44 (27)
Pre-frail, 4–5	660 (28)	304 (26)	196 (27)	106 (34)	54 (33)
Frail, 6–9	716 (30)	331 (29)	225 (31)	93 (30)	67 (41)
Comorbidities					
CCI, total score (0–33)	2 [1–3]	2 [0–3]	2 [1–3]	2 [1–3]	2 [1–3]
Lung disease	661 (28)	297 (26)	211 (29)	96 (31)	57 (35)
Diabetes with medication	727 (31)	360 (31)	231 (32)	93 (30)	43 (26)
Hypertension	1297 (55)	645 (56)	390 (53)	184 (60)	78 (47)
Myocardial infarction	409 (17)	197 (17)	131 (18)	59 (19)	22 (13)
Dementia	220 (9)	111 (10)	81 (11)	21 (7)	7 (4)
Smoking status					
Never	742 (43)	348 (43)	201 (39)	120 (49)	73 (49)
Former	815 (48)	379 (47)	283 (55)	101 (41)	52 (35)
Active	159 (9)	77 (10)	31 (6)	26 (11)	25 (17)

Data are mean ± SD, median [IQR] or n (%). Lung disease includes asthma, chronic obstructive pulmonary disease, interstitial lung disease and lung cancer. Number of missing values: All patients: 2 sex, 440 BMI, 43 living situation, 99 vaccinated, 383 CC, 3 diabetes with medication, 2 myocardial infarction, 2 dementia, 646 smoking status. First wave: 2 sex, 246 BMI, 28 living situation, 180 CCI, 3 diabetes with medication, 2 myocardial infarction, 2 dementia, 350 smoking status. Second wave: 125 BMI, 2 living situation, 117 CCI, 219 smoking status. Third wave: 60 BMI, 8 living situation, 40 vaccinated, 60 CCI, 62 smoking status. Fourth wave: 9 BMI, 5 living situation, 59 vaccinated, 26 CCI, 15 smoking status

*BMI* body mass index, *CCI* charlson comorbidity index, *NA* not applicable

*Vaccinated is defined as a patient who self-reportedly received at least one dose of a COVID-19 vaccine prior to admission (vaccinations were introduced after the second wave)

**Fig. 3:**
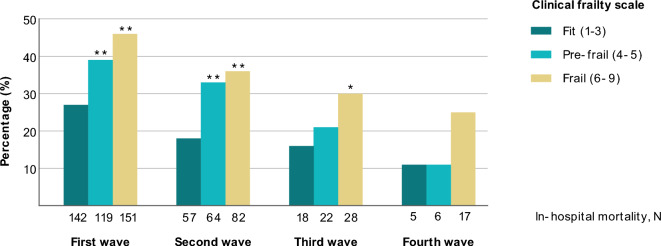
The association of frailty with in-hospital mortality stratified by COVID-19 wave. Comparisons with fit patients: *p value < 0.05, **p value < 0.001

In the first pandemic wave, in-hospital mortality was 46% in patients with frailty and 27% in fit patients. In-hospital mortality decreased in each subsequent wave to 25% in patients with frailty and 11% in fit patients in the fourth wave (Fig. 3). Compared to fit patients, patients with frailty had a higher risk of in-hospital mortality in the first (46% vs. 27%, *p* < 0.001), second (36% vs. 18%, *p* < 0.001), third (30% vs. 16%, *p* < 0.05) and fourth (25% vs. 11%, *p* > 0.05) wave.

**Fig. 4:**
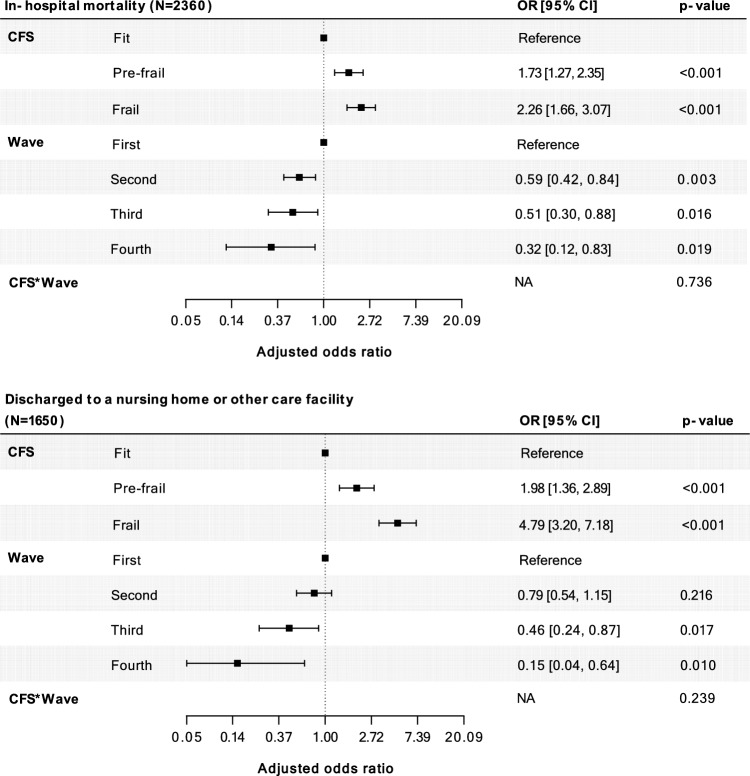
Multivariable adjusted associations of frailty with in-hospital mortality and discharge location. 95% CI = 95% confidence interval. Variables included in both models were: age, sex, clinical frailty scale, wave and clinical frailty scale*wave (interaction term)

Overall, after correction for age and sex, a higher risk of in-hospital mortality was found in frail (OR 2.26, 95% CI: 1.66–3.07) and pre-frail (OR 1.73, 95% CI: 1.27–2.35) patients, compared to fit patients (Fig. 4). The association of frailty with in-hospital mortality did not change over time (*p* for interaction = 0.74). Patients had a lower risk of in-hospital mortality in the second (OR 0.59, 95% CI: 0.42–0.84), third (OR 0.51, 95% CI: 0.30–0.88) and fourth (OR 0.32, 95% CI: 0.12–0.83) wave, compared to patients in the first wave. We performed a post hoc analysis of the association of frailty with in-hospital mortality with the selection of patients admitted to one of the four hospitals included in all waves (Appendix 4). The associations of frailty with in-hospital mortality followed a roughly similar pattern compared to our main findings. Discharge to a nursing home or other care facility was more frequent in frail (OR 4.79, 95% CI: 3.20–7.18) and pre-frail (OR 1.98, 95% CI: 1.36–2.89) patients, compared to fit patients. The association of frailty with discharge location did not change over time (*p* for interaction = 0.24).

To assess whether indicators of disease severity of hospitalised older people changed during the course of the pandemic, associations of frailty with indicators of disease severity were stratified by wave (Appendix 5). Compared to fit patients, frail and pre-frail patients had comparable vital signs (body temperature, respiratory rate, systolic blood pressure and saturation before oxygen therapy) in all four waves. With increasing levels of frailty, the duration of symptoms before admission was shorter in the first three waves (all *p* < 0.05). In the fourth wave, all patients had a median duration of symptoms before admission of 3 days. Compared to fit patients, frail and pre-frail patients mostly had comparable levels of thrombocytes, leucocytes, lymphocytes and sodium in all the different waves. Compared to fit patients, frail patients had lower levels of haemoglobin, C-reactive protein (CRP) and lactic acid dehydrogenase in the first two waves (all *p* < 0.001). In the third and fourth wave, frail patients still had lower levels of CRP compared to fit patients, although no longer significantly different. Compared to fit patients, pre-frail patients had higher median levels of creatinine in the first (99 vs. 89 μmol/L, *p* < 0.001), second (106 vs. 89 μmol/L, *p* < 0.001) and third (112 vs. 94 μmol/L, *p* < 0.05) wave. None of the observed associations of frailty with indicators of disease severity changed over time (*p* for interactions > 0.05).

To assess whether patient characteristics of older people with frailty changed during the course of the pandemic, differences in patient characteristics based on frailty were stratified by wave (Appendix 6). The median age of fit patients ranged from 76 to 77 years over the different waves. Compared to fit patients, frail patients were older in all waves with a median age ranging from 80 to 83 years (all *p* < 0.001). Compared to fit patients, pre-frail patients were older in the first three waves with a median age ranging from 79 to 80 years (all *p* < 0.001). Compared to fit patients, frail patients had a higher median CCI in the first, second and third wave (2 vs. 1 points, all *p* < 0.05). In the fourth wave, all patients had a median CCI of 2 points.

## Discussion

This study showed that older patients with frailty had a higher risk of in-hospital mortality throughout the entire COVID-19 pandemic, although overall in-hospital mortality risk decreased. Older patients with frailty showed only minor differences in disease severity at admission compared to fit patients reflected by comparable vital signs and minimal differences in laboratory findings.

We found that frailty remained an important risk factor of in-hospital mortality over the course of the COVID-19 pandemic, although in-hospital mortality decreased in each subsequent wave. A previous study of older hospitalised people with COVID-19 over five different waves in France also showed a decline in in-hospital mortality over time. However, mortality rates were 7–10% lower in that cohort in the first and second wave which could have been the reason why a less pronounced decline over time was observed compared to our study [15]. The higher rate of in-hospital mortality during the first two waves in our cohort is likely attributed to an incomparable patient selection because of differences in the duration of the pandemic waves and differences in organization of healthcare for acute ill older people in the Netherlands, compared to France. The overall observed decrease of in-hospital mortality over the two-year period in our study, likely reflects the previously reported effects of milder variants, such as Omicron, vaccinations and improved treatment options, such as dexamethasone and IL-6 inhibitors [16–20]. The lack of an interaction over time of the association between frailty and in-hospital mortality could indicate that improved treatment and prevention options have no frailty specific effect. Another explanation is that this study lacked statistical power to detect a frailty specific effect over time, as the sample size decreased in each subsequent wave. The lack of an interaction effect over time should be interpreted cautiously as treatment and prevention options were not directly compared between subgroups of patients based on their frailty status and remains a topic of further study. To our knowledge, we are the first to report associations of frailty with in-hospital mortality in hospitalised older people over a two-year period of the COVID-19 pandemic.

We found that patients with frailty showed only minor differences in disease severity at admission compared to fit patients reflected by comparable vital signs and minimal differences in laboratory findings. Interpretations of differences in disease severity should be made cautiously as frail patients presented themselves earlier in the course of infection. The earlier moment of presentation could itself be a sign of a more severe disease, but is more likely explained by reduced physical reserve or faster referrals by healthcare professionals for frail patients. Previous studies demonstrated that severe COVID-19 was associated with higher CRP levels in adults [21, 22]. In our study, frail patients had lower levels of CRP compared to fit patients. Frailty is known to be associated with chronic low-grade inflammation (inflammaging) which leads to chronically increased levels of certain biomarkers, such as CRP and IL-6, but may hamper the immune response in case of acute infection [23, 24]. This could explain the lower CRP levels. Frail patients have an increased susceptibility to infectious diseases, which could contribute to the relative increase of frail patients over time [25]. Another explanation could be a reserved referral policy that was applied for these patients in the Netherlands during the first two waves due to a high mortality risk and high pressure on the number of hospital beds. The observed association of frailty with in-hospital mortality during the entire COVID-19 pandemic, in combination with comparable disease severity and a relative increase of pre-frail and frail patients after the second wave, indicates that frail patients have a vulnerability to mortality that cannot be easily remedied despite milder variants and improved prevention and treatment options. A previous study found that frailty had a stronger association with disease outcomes than either age or comorbidities alone, which supports the theory of importance of underlying frailty in the course of COVID-19 infection [26]. Unsurprisingly, this vulnerability of frail patients is also reflected by the observed association of frailty with older age and comorbidities in our cohort. It is postulated that immunosenescence, besides inflammaging, attributes to this vulnerability, but this is currently not fully understood [24, 27].

Our findings indicate that despite milder variants over time and roughly comparable disease severity between frail and fit older patients, frail older people have an intrinsic vulnerability to mortality that cannot be easily remedied with timely initiation of hospitalisation and specific COVID-19 treatment options. These findings show the relevance of frailty as a risk factor in all stages of a pandemic. In the event of a new pandemic, frailty should be considered early on in government communication and policies and in prevention and treatment guidelines. The prognostic value of frailty in decision-making remains subject of further study.

This study has several limitations that need to be considered while interpreting the main findings of this study. The numbers of hospitals that could participate decreased over time, mainly due to increased individual workload for medical professionals during the COVID-19 pandemic and the lack of funding for scientific research. This resulted in a reduction of the sample size in each subsequent wave which affected statistical power over time and could potentially hamper generalizability of results found in the fourth wave. Another limitation is the lack of data on clinical deterioration during admission. Therefore, we could only evaluate disease severity at the time of admission. In addition, details on the numbers of patients with prospective versus retrospective assessment of frailty were not collected. To evaluate possible selection bias introduced by frailty measurements, we compared baseline characteristics between patients with an assessed CFS and CFS missing, which showed no relevant differences, indicating that selection bias was limited at most. Our study had several strengths. To our knowledge, this is the first study with an ongoing data collection during four waves of the COVID-19 pandemic over a 2 year period that systematically analysed associations of frailty in hospitalised older people. Other strengths of this study are the inclusion of both academic and peripheral hospitals across the Netherlands, the large sample size and the collection of a wide variety of variables.

In conclusion, frailty remained associated with a higher risk of in-hospital mortality throughout the entire COVID-19 pandemic, although overall in-hospital mortality decreased. These findings show the relevance of frailty as a risk factor in all stages of a pandemic, which indicates that frailty is important to consider in prevention and treatment guidelines for future pandemics.

## Supplementary Information

Below is the link to the electronic supplementary material.

Supplementary file1 (DOCX 106 KB)

## Data Availability

The data collected in this study are available upon reasonable request by contacting the principal investigator of this study (s.p.mooijaart@lumc.nl). Deidentified participant data and a data dictionary will be available with publication. Additional related documents are not available. A request should include a complete analysis plan with clear hypothesis and aim of the proposed study. To gain access, data requestors will need to sign a data access agreement after approval of the request.

## Abbreviations

IL-6: Interleukin-6
SARS-CoV-2: Severe acute respiratory syndrome coronavirus 2
PCR: Polymerase chain reaction
CCI: Charlson comorbidity index
SD: Standard deviation
IQR: Interquartile range
CRP: C-reactive protein

## References

[1] RIVM. Corona Dashboard. Available from: https://coronadashboard.rijksoverheid.nl/. last Accessed Sept 2023

[2] Chen Y, Klein SL, Garibaldi BT, Li H, Wu C, Osevala NM et al (2021) Aging in COVID-19: vulnerability, immunity and intervention. Ageing Res Rev 65:101205 33137510 10.1016/j.arr.2020.101205 PMC7604159

[3] Gallo Marin B, Aghagoli G, Lavine K, Yang L, Siff EJ, Chiang SS et al (2021) Predictors of COVID-19 severity: a literature review. Rev Med Virol 31(1):1–10 32845042 10.1002/rmv.2146 PMC7855377

[4] Pansarasa O, Pistono C, Davin A, Bordoni M, Mimmi MC, Guaita A et al (2019) Altered immune system in frailty: genetics and diet may influence inflammation. Ageing Res Rev 54:100935 31326616 10.1016/j.arr.2019.100935

[5] Fan Y, Li X, Zhang L, Wan S, Zhang L, Zhou F (2022) SARS-CoV-2 Omicron variant: recent progress and future perspectives. Signal Transduct Target Ther 7(1):141 35484110 10.1038/s41392-022-00997-x PMC9047469

[6] Forchette L, Sebastian W, Liu T (2021) A comprehensive review of COVID-19 virology, vaccines, variants, and therapeutics. Curr Med Sci 41(6):1037–1051 34241776 10.1007/s11596-021-2395-1 PMC8267225

[7] Flores-Vega VR, Monroy-Molina JV, Jimenez-Hernandez LE, Torres AG, Santos-Preciado JI, Rosales-Reyes R (2022) SARS-CoV-2: evolution and emergence of new viral variants. Viruses 14(4):653 35458383 10.3390/v14040653 PMC9025907

[8] Blomaard LC, van der Linden CMJ, van der Bol JM, Jansen SWM, Polinder-Bos HA, Willems HC et al (2021) Frailty is associated with in-hospital mortality in older hospitalised COVID-19 patients in the Netherlands: the COVID-OLD study. Age Ageing 50(3):631–640 33951156 10.1093/ageing/afab018 PMC7929372

[9] Smits RAL, Trompet S, van der Linden CMJ, van der Bol JM, Jansen SWM, Polinder-Bos HA et al (2022) Characteristics and outcomes of older patients hospitalised for COVID-19 in the first and second wave of the pandemic in The Netherlands: the COVID-OLD study. Age Ageing 51(3):afac048 35235650 10.1093/ageing/afac048 PMC8890695

[10] RIVM. De coronaprik. Available from: https://coronadashboard.rijksoverheid.nl/landelijk/de-coronaprik. last Accessed Sept 2023

[11] Charlson ME, Pompei P, Ales KL, MacKenzie CR (1987) A new method of classifying prognostic comorbidity in longitudinal studies: development and validation. J Chronic Dis 40(5):373–383 3558716 10.1016/0021-9681(87)90171-8

[12] Rockwood K, Song X, MacKnight C, Bergman H, Hogan DB, McDowell I et al (2005) A global clinical measure of fitness and frailty in elderly people. CMAJ 173(5):489–495 16129869 10.1503/cmaj.050051 PMC1188185

[13] Stille K, Temmel N, Hepp J, Herget-Rosenthal S (2020) Validation of the clinical frailty scale for retrospective use in acute care. Eur Geriatr Med 11(6):1009–1015 32770462 10.1007/s41999-020-00370-7

[14] Kay RS, Hughes M, Williamson TR, Hall AJ, Duckworth AD, Clement ND (2022) The clinical frailty scale can be used retrospectively to assess the frailty of patients with hip fracture: a validation study. Eur Geriatr Med 13(5):1101–1107 35987870 10.1007/s41999-022-00686-6 PMC9553782

[15] Thietart S, Rozes A, Tubach F, Marot S, Marcelin A-G, Raux M et al (2023) In-hospital mortality of older patients with COVID-19 throughout the epidemic waves in the great Paris area: a multicenter cohort study. BMC Geriatr 23(1):573 37723419 10.1186/s12877-023-04236-y PMC10507910

[16] Group RC, Horby P, Lim WS, Emberson JR, Mafham M, Bell JL et al (2021) Dexamethasone in hospitalized patients with covid-19. N Engl J Med 384(8):693–704 32678530 10.1056/NEJMoa2021436 PMC7383595

[17] Xu K, Wang Z, Qin M, Gao Y, Luo N, Xie W et al (2023) A systematic review and meta-analysis of the effectiveness and safety of COVID-19 vaccination in older adults. Front Immunol 14:1113156 36936964 10.3389/fimmu.2023.1113156 PMC10020204

[18] Shankar-Hari M, Vale CL, Godolphin PJ, Fisher D, Higgins JPT, Spiga F et al (2021) Association between administration of IL-6 antagonists and mortality among patients hospitalized for COVID-19: a meta-analysis. JAMA 326(6):499–518 34228774 10.1001/jama.2021.11330 PMC8261689

[19] Nealon J, Cowling BJ (2022) Omicron severity: milder but not mild. Lancet 399(10323):412–413 35065007 10.1016/S0140-6736(22)00056-3 PMC8769661

[20] Wang C, Liu B, Zhang S, Huang N, Zhao T, Lu QB et al (2023) Differences in incidence and fatality of COVID-19 by SARS-CoV-2 Omicron variant versus Delta variant in relation to vaccine coverage: a world-wide review. J Med Virol 95(1):e28118 36056540 10.1002/jmv.28118 PMC9537802

[21] Zeng F, Huang Y, Guo Y, Yin M, Chen X, Xiao L et al (2020) Association of inflammatory markers with the severity of COVID-19: a meta-analysis. Int J Infect Dis 96:467–474 32425643 10.1016/j.ijid.2020.05.055 PMC7233226

[22] Chen W, Zheng KI, Liu S, Yan Z, Xu C, Qiao Z (2020) Plasma CRP level is positively associated with the severity of COVID-19. Ann Clin Microbiol Antimicrob 19(1):18 32414383 10.1186/s12941-020-00362-2 PMC7227180

[23] Velissaris D, Pantzaris N, Koniari I, Koutsogiannis N, Karamouzos V, Kotroni I et al (2017) C-reactive protein and frailty in the elderly: a literature review. J Clin Med Res 9(6):461–465 28496545 10.14740/jocmr2959w PMC5412518

[24] Witkowski JM, Fulop T, Bryl E (2022) Immunosenescence and COVID-19. Mech Ageing Dev 204:111672 35378106 10.1016/j.mad.2022.111672 PMC8975602

[25] Vetrano DL, Triolo F, Maggi S, Malley R, Jackson TA, Poscia A et al (2021) Fostering healthy aging: the interdependency of infections, immunity and frailty. Ageing Res Rev 69:101351 33971332 10.1016/j.arr.2021.101351 PMC9588151

[26] Hewitt J, Carter B, Vilches-Moraga A, Quinn TJ, Braude P, Verduri A et al (2020) The effect of frailty on survival in patients with COVID-19 (COPE): a multicentre, European, observational cohort study. Lancet Public Health 5(8):e444–e451 32619408 10.1016/S2468-2667(20)30146-8 PMC7326416

[27] Wang Y, Dong C, Han Y, Gu Z, Sun C (2022) Immunosenescence, aging and successful aging. Front Immunol 13:942796 35983061 10.3389/fimmu.2022.942796 PMC9379926

