# Supplementary material for: Preparing for future pandemics: frailty associates with mortality in hospitalised older people during the entire COVID-19 pandemic, a Dutch multicentre cohort study
Source: Eur Geriatr Med. 2024 Jun 7;15(4):951–9. doi: 10.1007/s41999-024-01001-1 (PMC11377458; doi:10.1007/s41999-024-01001-1)
Supplement: Supplementary file 1 — Supplementary file1 (DOCX 106 KB) [file 41999_2024_1001_MOESM1_ESM.docx]

**Supplementary Appendix**

Preparing for future pandemics: frailty associates with mortality in hospitalised older people during the entire COVID-19 pandemic, a Dutch multicentre cohort study.

*European Geriatric Medicine*

**Authors**

Bas F.M. van Raaij, Raymond Noordam, Rosalinde A.L. Smits, Veerle M.G.T.H. van der Klei, Steffy W.M. Jansen, Carolien M.J. van der Linden, Harmke A. Polinder-Bos, Julia Minnema, Lisanne Tap, Jessica M. van der Bol, Esther M.M. van de Glind, Hanna C. Willems, Floor J.A. van Deudekom, Rikje Ruiter, Barbara C. van Munster, Sarah H.M. Robben, Henrike J. Schouten, Dennis G. Barten, Jacinta A. Lucke, Geeske M.E.E. Peeters, Stella Trompet, Yvonne M. Drewes, Frederiek van den Bos, Jacobijn Gussekloo, Simon P. Mooijaart, on behalf of the COOP study group.

| **Contents** |  |
| --- | --- |
| Appendix 1. List of participating hospitals . . . . . . . . . . . . . . . . . . . . . . . . . . . . . . . . . . . . . . . . . . . . . . . . . . . . | 3 |
| Appendix 2. Number of inclusions per hospital for each pandemic wave . . . . . . . . . . . . . . . . . . . . . . . . . . . | 4 |
| Appendix 3. Comparison of patients with a recorded frailty status and patients with a missing frailty status . . . . . . . . . . . . . . . . . . . . . . . . . . . . . . . . . . . . . . . . . . . . . . . . . . . . . . . . . . . . . . . . . . . . . . . . . . . . . . . . . . | 5 |
| Appendix 4. The association of frailty with in-hospital mortality stratified by COVID-19 wave of patients admitted to one of four hospitals included in all waves . . . . . . . . . . . . . . . . . . . . . . . . . . . . . . . . . . . . . . . . . . | 6 |
| Appendix 5. The associations of frailty with indicators of disease severity stratified by COVID-19 wave . . | 7 |
| Appendix 6. Differences in patients characteristics based on frailty stratified by COVID-19 wave . . . . . . . | 9 |

**Appendix 1. List of participating hospitals**

| **Hospital** | **Location** |
| --- | --- |
| Alrijne | Leiderdorp |
| Amsterdam University Medical Center | Amsterdam |
| Catharina Hospital | Eindhoven |
| Deventer Hospital | Deventer |
| Elisabeth-TweeSteden | Tilburg |
| Erasmus Medical Center | Rotterdam |
| Gelre | Apeldoorn and Zutphen |
| Isala | Zwolle |
| Leiden University Medical Center | Leiden |
| Maasstad Hospital | Rotterdam |
| Medical Center Leeuwarden | Leeuwarden |
| OLVG | Amsterdam |
| Reinier de Graaf | Delft |
| Spaarne | Haarlem |
| St. Jansdal | Harderwijk |
| University Medical Center Groningen | Groningen |
| VieCuri Medical Center | Venlo |
| Zaans Medical Center | Zaandam |
| ZGT | Almelo |

**Appendix 2. Number of inclusions per hospital for each pandemic wave**


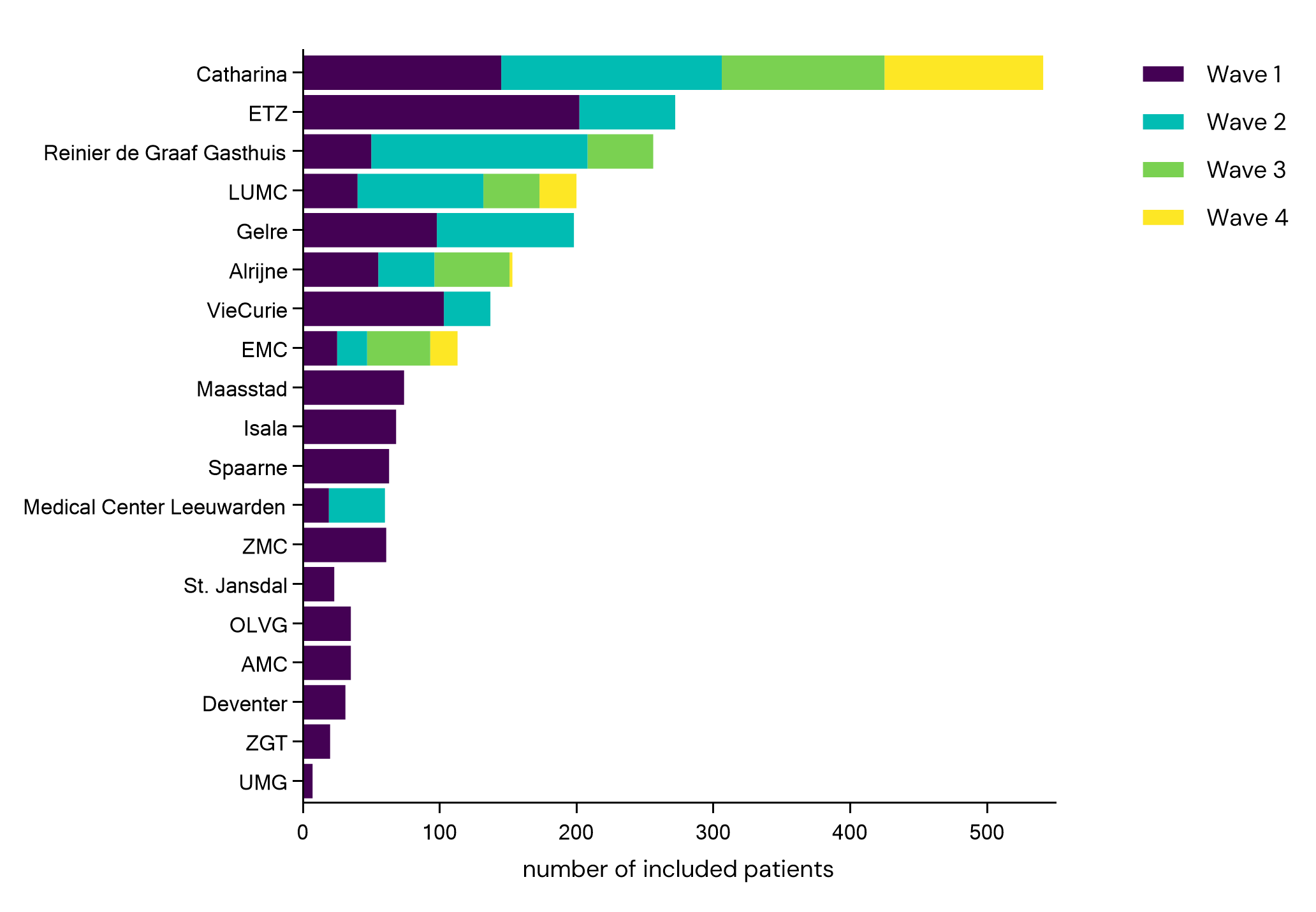


**Appendix 3. Comparison of patients with a recorded frailty status and patients with a missing frailty status.**

| Characteristic | Clinical frailty scale | |  |
| --- | --- | --- | --- |
|  | Assessed (N=2362) | Missing (N=705) |  |
| Age, years | 79 [75-84] | 78 [74-83] |  |
| Sex, men | 1421 (60) | 440 (62) |  |
| BMI, kg/m^2^ | 26.9 ±5.0 | 27.3 ± 4.9 |  |
| Living situation |  |  |  |
| Home | 2075 (90) | 598 (91) |  |
| Nursing home | 220 (10) | 47 (7) |  |
| Other | 24 (1) | 12 (2) |  |
| Vaccinated* | 290 (77) | 47 (71) |  |
| Comorbidities |  |  |  |
| CCI, total score (0-33) | 2 [1-3] | 2 [1-3] |  |
| Lung disease | 661 (28) | 211 (30) |  |
| Diabetes with medication | 727 (31) | 222 (32) |  |
| Hypertension | 1297 (55) | 389 (55) |  |
| Myocardial infarction | 409 (17) | 146 (21) |  |
| Dementia | 220 (9) | 32 (5) |  |
| Smoking status |  |  |  |
| Never | 742 (43) | 225 (45) |  |
| Former | 815 (48) | 244 (49) |  |
| Active | 159 (9) | 28 (6) |  |

Data are mean ± SD, median [IQR] or n (%). BMI=body mass index. CCI=charlson comorbidity index.

Lung disease includes asthma, chronic obstructive pulmonary disease, interstitial lung disease and lungcancer.

* Vaccinated is defined as a patient who self-reportedly received at least one dose of a COVID-19 vaccine prior to admission.

Number of missing values:

Clinicial frailty status assessed: 2 sex, 440 BMI, 43 living situation, 99 vaccinated, 383 CCI, 3 diabetes with medication, 2 myocardial infarction, 2 dementia, 646 smoking status.

Clinical frailty status missing: 139 BMI, 48 living situation, 30 vaccinated, 109 CCI, 2 hypertension, 208 smoking status.

**Appendix 4. The association of frailty with in-hospital mortality stratified by COVID-19 wave of patients admitted to one of four hospitals included in all waves.**

|  | **First wave** | | |  | **Second wave** | | |  | **Third wave** | | |  | **Fourth wave** | | |  | **P-value for interaction** |
| --- | --- | --- | --- | --- | --- | --- | --- | --- | --- | --- | --- | --- | --- | --- | --- | --- | --- |
|  | **Fit**  **(N=124)** | **Pre-frail (N=64)** | **Frail**  **(N=77)** |  | **Fit**  **(N=125)** | **Pre-frail (N=79)** | **Frail**  **(N=112)** |  | **Fit**  **(N=91)** | **Pre-frail (N=92)** | **Frail**  **(N=78)** |  | **Fit**  **(N=44)** | **Pre-frail (N=54)** | **Frail**  **(N=67)** |  |  |
| In-hospital mortality, n (%) | 40 (32) | 18 (28) | 45 (58)** |  | 16 (13) | 20 (25)* | 40 (36)** |  | 15 (17) | 18 (20) | 23 (30)* |  | 5 (11) | 6 (11) | 17 (25) |  | 0.423 |

Comparisons with fit patients: * p value < 0.05, ** p-value <0.001

**Appendix 5. The associations of frailty with indicators of disease severity stratified by COVID-19 wave.**

|  | **First wave** | | |  | **Second wave** | | |  | **Third wave** | | |  | **Fourth wave** | | |  | **P-value for interaction** |
| --- | --- | --- | --- | --- | --- | --- | --- | --- | --- | --- | --- | --- | --- | --- | --- | --- | --- |
|  | **Fit**  **(N=519)** | **Pre-frail (N=304)** | **Frail**  **(N=331)** |  | **Fit**  **(N=313)** | **Pre-frail (N=196)** | **Frail**  **(N=224)** |  | **Fit**  **(N=110)** | **Pre-frail (N=106)** | **Frail**  **(N=93)** |  | **Fit**  **(N=44)** | **Pre-frail (N=54)** | **Frail**  **(N=67)** |  |  |
| DSBA, days | 7  [5-11] | 7**  [3-11] | 4**  [2-7] |  | 7  [4-10] | 5*  [2-8] | 4**  [1-7] |  | 7  [4-10] | 5*  [1-8] | 3**  [1-7] |  | 3  [1-9] | 3  [0-8] | 3  [1-6] |  | 0.063 |
| Temp, °C | 37.8±1.1 | 37.7±1.1 | 37.6±1.1 |  | 37.8±1.1 | 37.8±1.0 | 37.7±1.0 |  | 37.7±1.0 | 37.6±1.0 | 37.7±1.1 |  | 37.2±1.2 | 37.2±1.1 | 37.1±1.5 |  | 0.835 |
| RR, breaths/min | 20  [18-26] | 22  [18-25] | 20  [18-25] |  | 21  [18-27] | 22  [18-26] | 21  [18-27] |  | 20  [16-26] | 20  [16-25] | 22  [18-28] |  | 18  [14-21] | 20*  [16-25] | 20  [16-24] |  | 0.878 |
| SBP, mmHg | 139±23 | 137±23 | 139±24 |  | 139±25 | 137±24 | 138±28 |  | 141±27 | 138±25 | 139±27 |  | 145±30 | 140±30 | 143±33 |  | 0.851 |
| SBOT, % | 92  [89-95] | 93  [89-95] | 94  [88-96] |  | 93  [90-96] | 94  [91-97] | 94  [90-97] |  | 93  [90-95] | 94  [89-96] | 93  [90-95] |  | 95  [91-97] | 95  [92-98] | 95  [92-97] |  | 0.741 |
| Oxygen therapy, L/min | 3  [2-5] | 3  [2-4] | 2*  [0-4] |  | 2  [0-5] | 2*  [0-4] | 2  [0-4] |  | 3  [1-6] | 2  [0-5] | 2  [0-5] |  | 0  [0-3] | 2  [0-4] | 1  [0-3] |  | 0.695 |
| Haemoglobin, mmol/L | 8.4±1.2 | 8.0±1.2** | 7.7±1.2** |  | 8.2±1.2 | 8.0±1.3 | 7.8±1.2** |  | 8.3±1.1 | 8.0±1.2 | 8.0±1.3 |  | 8.0±1.3 | 7.7±1.3 | 7.4±1.8* |  | 0.108 |
| Thrombocytes, 10^9^/L | 221±113 | 207±85 | 209±88 |  | 206±91 | 205±109 | 212±84 |  | 209±90 | 221±93 | 211±129 |  | 240±97 | 214±75 | 229±78 |  | 0.232 |
| Leucocytes,  10^9^/L | 7.9±4.7 | 8.8±12.1 | 8.4±7.3 |  | 7.3±3.7 | 7.4±8.6 | 7.6±3.9 |  | 7.8±3.7 | 9.4±13.4 | 9.0±5.8 |  | 9.5±4.6 | 9.1±4.8 | 9.5±4.0 |  | 0.725 |
| Lymphocytes, 10^9^/L | 0.90  [0.60-1.40] | 1.00*  [0.64-1.60] | 0.94*  [0.58-1.62] |  | 0.78  [0.51-1.10] | 0.80  [0.52-1.10] | 0.79  [0.60-1.01] |  | 0.88  [0.60-1.20] | 0.83  [0.55-1.20] | 0.80  [0.53-1.04] |  | 1.10  [0.57-1.51] | 0.72  [0.40-1.12] | 0.93  [0.51-1.33] |  | 0.114 |
| CRP, mg/L | 94  [49-152] | 76*  [38-138] | 63**  [31-111] |  | 79  [43-149] | 67*  [30-116] | 62**  [25-111] |  | 84  [37-159] | 77  [37-140] | 53  [27-111] |  | 54  [16-130] | 55  [16-100] | 33  [12-95] |  | 0.380 |
| LDH, U/L | 335  [258-439] | 306  [248-423] | 296**  [235-388] |  | 332  [258-436] | 291*  [241-379] | 288**  [229-362] |  | 341  [252-444] | 287*  [232-369] | 284  [223-416] |  | 249  [185-319] | 243  [195-334] | 254  [210-315] |  | 0.689 |
| Creatinin, µmol/L | 89  [75-112] | 99**  [76-142] | 92  [70-140] |  | 89  [72-120] | 106**  [84-147] | 102*  [76-139] |  | 94  [72-122] | 112*  [83-138] | 98  [70-134] |  | 94  [71-119] | 96  [72-142] | 99  [71-141] |  | 0.695 |
| Sodium, mmol/L | 135±4 | 136±5 | 137±6** |  | 135±4 | 136±4 | 137±5* |  | 134±5 | 137±5* | 136±4 |  | 136±4 | 137±4 | 138±5 |  | 0.427 |

Comparisons with fit patients: * p value < 0.05, ** p-value <0.001.

Data are mean ± SD or median [IQR]. DSBA=duration of symptoms before admission. Temp=body temperature. RR=respiratory rate. SBP=systolic blood pressure. SBOT=saturation before oxygen therapy. CRP=c-reactive protein. LDH= lactic acid dehydrogenase.

Reference values for laboratory findings:

Haemoglobin 8.5-11.0; Thrombocytes 150-400; Leucocytes 4.00-10.00; Lymphocytes 1.00-3.50; CRP <5.0; LDH <248; creatinin 64-104; Sodium 136-145.

Number of missing values:

First wave: 119 DSBA, 55 Temp, 70 RR, 58 SBP, 362 SBOT, 124 oxygen therapy, 10 haemoglobin, 94 thrombocytes, 64 leucocytes, 188 lymphocytes, 65 CRP, 219 LDH, 60 creatinin, 13 sodium.

Second wave: 52 DSBA, 17 Temp, 30 RR, 12 SBP, 148 SBOT, 42 oxygen therapy, 16 haemoglobin, 25 thrombocytes, 27 leucocytes, 107 lymphocytes, 18 CRP, 88 LDH, 12 creatinin, 20 sodium.

Third wave: 23 DSBA, 10 Temp, 11 RR, 2 SBP, 110 SBOT, 25 oxygen therapy, 6 haemoglobin, 11 thrombocytes, 5 leucocytes, 41 lymphocytes, 5 CRP, 36 LDH, 2 creatinin, 8 sodium.

Fourth wave: 28 DSBA, 4 Temp, 17 RR, 41 SBOT, 7 oxygen therapy, 2 haemoglobin, 3 thrombocytes, 2 leucocytes, 32 lymphocytes, 5 CRP, 35 LDH, 1 creatinin, 3 sodium

**Appendix 6. Differences in patients characteristics based on frailty stratified by COVID-19 wave.**

| **Characteristic** | **First wave** | | |  | **Second wave** | | |  | **Third wave** | | |  | **Fourth wave** | | |
| --- | --- | --- | --- | --- | --- | --- | --- | --- | --- | --- | --- | --- | --- | --- | --- |
|  | **Fit**  **(N=519)** | **Pre-frail (N=304)** | **Frail**  **(N=331)** |  | **Fit**  **(N=313)** | **Pre-frail (N=196)** | **Frail**  **(N=224)** |  | **Fit**  **(N=110)** | **Pre-frail (N=106)** | **Frail**  **(N=93)** |  | **Fit**  **(N=44)** | **Pre-frail (N=54)** | **Frail**  **(N=67)** |
| Age, years | 76  [73-81] | 79  [75-84]** | 82  [78-87]** |  | 77  [73-82] | 80  [75-86]** | 83  [78-87]** |  | 77  [73-81] | 80  [75-85]** | 80  [76-86]** |  | 76  [74-82] | 80  [75-83] | 82  [77-87]** |
| Sex, men | 359 (69) | 174 (57)** | 156 (47)** |  | 203 (65) | 124 (63) | 119 (53)* |  | 76 (69) | 67 (63) | 51 (55)* |  | 33 (75) | 29 (54)* | 30 (45)* |
| BMI, kg/m^2^ | 26.9±4.1 | 28.1±5.5* | 26.8±4.9 |  | 26.7±4.7 | 27.2±4.9 | 26.7±5.8 |  | 26.6±4.5 | 27.0±5.6 | 26.4±6.3 |  | 26.1±4.0 | 25.7±5.1 | 26.5±6.2 |
| Living situation |  |  |  |  |  |  |  |  |  |  |  |  |  |  |  |
| Home | 501 (99) | 279 (95)** | 228 (70)** |  | 303 (97) | 186 (95) | 152 (68)** |  | 108 (100) | 98 (95)* | 72 (80)** |  | 43 (100) | 50 (93) | 55 (87)* |
| Nursing home | 1 (0) | 13 (4)** | 89 (27)** |  | 8 (3) | 6 (3) | 70 (31)** |  | 0 (0) | 5 (5)* | 18 (20)** |  | 0 (0) | 3 (6) | 7 (11)* |
| Other | 4 (1) | 2 (1)** | 9 (3)** |  | 1 (0) | 3 (2) | 3 (1)** |  | 0 (0) | 0 (0)* | 0 (0)** |  | 0 (0) | 1 (2) | 1 (2)* |
| Vaccinated^a^ | NA | NA | NA |  | NA | NA | NA |  | 66 (70) | 79 (86)* | 67 (82) |  | 16 (70) | 25 (76) | 37 (74) |
| **Comorbidities** |  |  |  |  |  |  |  |  |  |  |  |  |  |  |  |
| CCI, total score | 1 [0-2] | 2 [1-3]** | 2 [1-3]** |  | 1 [0-2] | 2 [1-3]** | 2 [1-3]** |  | 1 [0-3] | 2 [1-3] | 2 [1-3]* |  | 2 [1-2] | 2 [1-4] | 2 [1-3] |
| Lung disease | 102 (20) | 103 (34)** | 92 (28)* |  | 84 (27) | 55 (28) | 72 (32) |  | 20 (18) | 39 (37)* | 37 (40)** |  | 16 (36) | 20 (37) | 21 (31) |
| Diabetes with medication | 133 (26) | 106 (35)* | 121 (37)** |  | 76 (24) | 71 (36)* | 84 (37)* |  | 33 (30) | 32 (30) | 28 (30) |  | 9 (21) | 13 (24) | 21 (31) |
| Hypertension | 254 (49) | 193 (64)** | 198 (60)* |  | 160 (51) | 110 (56) | 120 (53) |  | 59 (54) | 65 (61) | 60 (65) |  | 15 (34) | 26 (48) | 37 (55)* |
| Myocardial infarction | 76 (15) | 67 (22)* | 54 (16) |  | 45 (14) | 45 (23)* | 41 (18) |  | 17 (16) | 19 (18) | 23 (25) |  | 7 (16) | 5 (9) | 10 (15) |
| Dementia | 1 (0) | 16 (5)** | 94 (28)** |  | 4 (1) | 12 (6)* | 65 (29)** |  | 1 (1) | 4 (4) | 16 (17)** |  | 0 (0) | 1 (2) | 6 (9) |
| Smoking status |  |  |  |  |  |  |  |  |  |  |  |  |  |  |  |
| Never | 152 (42) | 84 (40) | 112 (48)* |  | 86 (39) | 47 (36) | 68 (42) |  | 49 (56) | 35 (42) | 36 (47)* |  | 22 (56) | 19 (40) | 32 (51) |
| Former | 178 (49) | 107 (51) | 94 (40)* |  | 125 (56) | 78 (59) | 80 (50) |  | 33 (38) | 44 (52) | 24 (32)* |  | 12 (31) | 18 (38) | 22 (35) |
| Active | 30 (8) | 18 (9) | 29 (12)* |  | 11 (5) | 7 (5) | 13 (8) |  | 5 (6) | 5 (6) | 16 (21)* |  | 5 (13) | 11 (23) | 9 (14) |

Comparisons with fit patients: * p value < 0.05, ** p-value <0.001.

Data are mean ± SD, median [IQR] or n (%). BMI=body mass index. CCI=charlson comorbidity index. NA=not applicable.

Lung disease includes asthma, chronic obstructive pulmonary disease, interstitial lung disease and lungcancer.

a. Vaccinated is defined as a patient who self-reportedly received at least one dose of a COVID-19 vaccine prior to admission (vaccinations were introduced after the second wave).

Number of missing values:

First wave: 2 sex, 246 BMI, 28 living situation, 180 CCI, 3 diabetes with medication, 2 myocardial infarction, 2 dementia, 350 smoking status.

Second wave: 125 BMI, 2 living situation, 117 CCI, 219 smoking status.

Third wave: 60 BMI, 8 living situation, 40 vaccinated, 60 CCI, 62 smoking status.

Fourth wave: 9 BMI, 5 living situation, 59 vaccinated, 26 CCI, 15 smoking status.
